# Diagnostic performance of intracystic carcinoembryonic antigen (CEA) versus glucose in differentiation of mucinous and non-mucinous pancreatic cysts

**DOI:** 10.3389/pore.2024.1611881

**Published:** 2024-10-10

**Authors:** György Gyimesi, Bánk Keczer, Péter Rein, Miklós Horváth, Ákos Szűcs, Tamás Marjai, Attila Szijártó, István Hritz

**Affiliations:** ^1^ School of Doctoral Studies, Semmelweis University, Budapest, Hungary; ^2^ Department of Gastroenterology, Spital Thurgau AG, Münsterlingen, Switzerland; ^3^ Department of Surgery, Transplantation and Gastroenterology, Semmelweis University, Budapest, Hungary; ^4^ Department of Surgery, Transplantation and Gastroenterology, Division of Interventional Gastroenterology, Semmelweis University, Budapest, Hungary

**Keywords:** pancreatic cyst, endosonography, FNA, intracystic CEA, intracystic glucose

## Abstract

**Background and Objectives:**

Pancreatic cysts have various potential for malignant transformation. Differentiating mucinous from non-mucinous cysts is crucial to make the right decision about further management, since mucinous cysts carry the risk of malignancy. Using endoscopic ultrasound (EUS) guided fine needle aspiration to determine intracystic carcinoembryonic antigen (CEA) levels is the recommended method for identifying mucinous cysts, although intracystic glucose assessment has also proved to be an effective tool. This study aims to compare the diagnostic performance of intracystic glucose and CEA in distinguishing between mucinous and non-mucinous pancreatic cystic lesions.

**Methods:**

In this single center study, we prospectively collected and analyzed the data of 91 consecutive patients who underwent endoscopic ultrasound (EUS)-guided fine-needle aspiration (FNA) with cytological analysis and measurement of intracystic CEA and glucose levels. The cyst type was classified based on radiological and EUS morphology, string sign, CEA, cytological and histological findings in resected cases. The diagnosis was established retrospectively by three experienced gastroenterologists blinded for glucose level in cases without definitive cytology or histology. We calculated the sensitivity, specificity, the positive- and negative predictive value of glucose and CEA respectively, and compared the two methods.

**Results:**

The sensitivity of intracystic glucose versus CEA proved to be 96.2% vs. 69.2% in identifying mucinous cysts, while the specificity of glucose was shown to be 79.5%, compared to 100% for CEA.

**Conclusion:**

Intracystic glucose is a sensitive, easily accessible biomarker in identifying mucinous pancreatic cysts, however, the specificity is lower compared to CEA. The measurement of intracystic glucose level could help in decision-making in daily clinical practice, however the diagnostic performance of the method remains inferior to “through-the-needle” techniques, such as confocal laser endomicroscopy and Moray forceps biopsy.

## Introduction

The widespread use of abdominal ultrasound and cross-sectional imaging with computed tomography (CT) and magnetic resonance imaging (MRI) has led to an increased diagnosis of asymptomatic pancreatic cysts. Studies reported a detection rate of 0.7%–36% in asymptomatic populations [1, 2]. Since pancreatic cysts have various potential for malignant transformation, it is of great importance to differentiate between the different types of cystic lesions.

Unfortunately, there is no single method or combination of diagnostic techniques which would grant a definitive diagnosis. This can be verified only after surgical resection or by positive intracystic fluid cytology for adenocarcinoma or neuroendocrine tumor.

In general, to guide the patient’s management, we need to utilize the patient’s previous history (e.g., pancreatitis), imaging modalities (such as CT, MRI) and evaluation of intracystic fluid samples obtained by EUS-guided fine needle aspiration (FNA). It is crucial to differentiate between mucinous and non-mucinous cysts since mucinous lesions are potentially malignant and require surgical resection or long-term follow up according to current international guidelines [3–5].

The accuracy of CT/Magnetic Resonance Cholangiopancreatography (MRCP) in determining a definitive diagnosis is merely about 50% [6, 7]. EUS-FNA, with the possibility of obtaining intracystic fluid for analysis of intracystic cytology and biomarkers, plays an outstanding role in diagnosing pancreatic cystic lesions. Cytology has a high specificity but a low sensitivity in detecting malignancy, and its role in differentiating cysts is limited. In three current meta-analyses of differentiating mucinous from non-mucinous cysts, the sensitivity and specificity of intracystic CEA (cut-off value 192 ng/mL) was shown to be 56%–67% and 80%–96%, while the sensitivity and specificity of glucose (cut-off value 50 mg/dL) was 91% and 75%–86% [8–10], respectively.

In our present study, we evaluated the sensitivity, specificity, diagnostic accuracy and positive and negative predictive value of CEA and glucose in the differentiation of mucinous from non-mucinous cystic lesions.

## Methods and materials

This study was approved by the Ethics Committee of the Semmelweis University of Budapest (Ethical approval number: 1121-1/2020/EKU). All patients provided informed consent prior to the procedure for obtaining intracystic fluid by EUS-FNA. The clinical records, EUS, CT/MRI reports and images, laboratory results, cytology, pathology and surgical reports included are all well documented and reliable. We collected the data in a prospective manner between September 2020 and December 2023. The endosonography scans were performed by two expert endoscopists (I.H. and M.H.), each with an overall experience of more than 1000 pancreatic EUS. A UCT-180 linear echoendoscope and an EU-ME2 Premium endoscopic ultrasound processor (Olympus GmbH) were used to visualize the EUS-morphology of the lesions. All the cysts were punctured with a 19/22/25 G EchoTip Ultra FNA needle (Cook Co., Boston, United States) and a 10 mL syringe vacuum suction was applied. “String sign” was documented in 52 cases (57.1%) and the collected fluid samples were used for cytological analysis and for assessment of intracystic amylase, CEA and glucose levels. Only cysts with successfully measured both CEA and glucose level were included in the study. No complications were observed in relation to the EUS-FNA. The specific cyst type was defined based on previous clinical follow-up, cyst morphology on cross-sectional imaging and EUS, “string sign,” intracystic amylase level, cytological characteristics, definitive cytology (5 cases, 5.5%) and post-operative histology (13 cases, 14.3%) in resected cases. The CEA cut-off level for mucinous cysts was determined by > 192 ng/mL. The diagnosis was established retrospectively by three experienced gastroenterologists (GG, IH, and MH) blinded for glucose level in cases without definitive cytology or histology. The study cohort consisted of patients who underwent EUS FNA and cyst fluid analysis, where the cyst type was unclear and where the results were likely to alter the management. Cysts with obvious morphological signs of malignancy were not included in the study. Patients were excluded if a consensual final diagnosis between the three experts could not be established. The mean follow-up time was 921 days.

### EUS, CT/MRI morphology

During EUS, we assessed and documented the localization, size, number of cysts, lobularity, cyst wall thickness, septa, nodules, solid masses associated with the cyst, pancreatic duct diameter, communication of the cyst with the pancreatic duct and lymphadenopathy. Multidetector computed tomography (MDCT) or MRI/MRCP were also performed in each patient. The same morphological criteria were used during cross-sectional imaging to define the cyst type as for EUS. All cysts were initially assessed by three experienced pancreatologists (IH, MH, and GG) and a consensual decision was made about the suspected diagnosis. Cases with a suspicion of a premalignant or malignant lesion were also discussed in an interdisciplinary pancreatic board meeting (e.g., Pancreas Team).

### String sign

The “String Sign” test involved placing a sample of intracystic fluid between the index finger and thumb, and measuring the distance before the string broke when separating the fingers at a minimum length of 3–4 mm and lasting for at least 1 second.

### Cytology

Cytological analysis was performed using haematoxylin-eosin and Papanicolaou staining. The presence of malignant cells, cells with atypia, mucin-containing cells or glycogen-containing cells was assessed. Presence of extracellular mucin and inflammatory cells was also documented.

### CEA and glucose

The obtained fluid samples were immediately transferred to the laboratory and measurements took place within 4 h. Both CEA (by electro-chemiluminescence using an enzyme-labelled sandwich immunoassay) and glucose (by spectrophotometric assessment using Hexokinase) measurements were performed by the Clinical Laboratory of Semmelweis University of Budapest. Regarding intracystic CEA, we applied the standardized cut-off value of 192 ng/mL according to the classic study of Brugge et al. [11], and cut-off for the glucose level was defined as 50 mg/dL (2.8 mmol/L) based on the studies of Zikos et al. [12], to differentiate between mucinous and non-mucinous cysts.

### Amylase

We applied amylase level under 250 U/L as a criterion to exclude pseudocysts.

### Statistical analysis

Data collection, evaluation, and figure generation were performed using the IBM SPSS Statistics 25, GraphPad Prism and Microsoft Office Excel software. Normality tests (Anderson–Darling, D’Agostino and Pearson, Shapiro–Wilk, Kolmogorov–Smirnov) were performed to determine the normality level of the samples. In instances where a normal distribution was observed, independent two-sample *t*-test was employed for intergroup comparisons. For continuous variables that deviated from a normal distribution, non-parametric tests were utilized for comparative investigations. Categorical variables were characterized by specifying the number of elements in the corresponding category and calculating the percentage distribution. For statistical analyses, we utilized the χ^2^ test or, in cases of low (less than 5) expected values, the Fisher’s exact test. Continuous variables were presented as mean ± standard deviation (SD) or median with range, as appropriate for the data distribution. All statistical tests were conducted assuming a two-tailed distribution, with a significance threshold set at *p* < 0.05.

## Results

In total, pancreatic cystic fluid was obtained from 97 patients and analyzed for cytology, amylase, CEA and glucose content. 4 cases were excluded from the analysis because of obvious signs of infected cyst fluid (cytological or microbiological testing) and 2 other cases because of significant blood content in the fluid (by macroscopic assessment), since these conditions may lead to false glucose and CEA levels, respectively.

Of the 91 patients, 28 (30.8%) were male and 63 (69.2%) were female. The median age was 72.4 years (SD 12.18) in the mucinous group and 56.4 years (SD 15.48) in the non-mucinous group (*p* < 0.0001). The median cyst diameter on endosonography was 28 mm in the mucinous group and 38 mm in the non-mucinous group (p 0.054). 43 cysts were unilocular and 48 were multilocular. 28 cysts were localized in the uncinate process and in the pancreatic head, 59 in the body and 4 in the tail. The patient and cyst characteristics are presented in Table 1.

**TABLE 1:** Population demographics and cyst characteristics.

	Mucinous	Non-Mucinous	*p-*value
Gender			
Male, n (%)	18 (35)	10 (26)	
Female, n (%)	34 (65)	29 (74)	
Age in years, median (IQR)	72.4 (76.58–65.95)	56.4 (71.2–42.6)	*<0.0001*

Cyst characteristics	Mucinous	Non-Mucinous	*p-*value
Localization of the largest cyst, n, (%)			
Uncinate process	3 (6)	0 (0)	
Head	12 (23)	13 (33)	
Genu of pancreas	8 (15)	8 (21)	
Body	16 (30)	14 (36)	
Body-tail border	11 (21)	2 (5)	
Tail	2 (4)	2 (5)	
Largest cyst diameter in mm, median (95% CI)	28 (24–32)	38 (25–41)	*0.054*
Unilocular cyst, n (%)	24 (46)	19 (49)	
Multilocular cyst, n (%)	28 (54)	20 (51)	

13 cases (14.3%) were diagnosed by post-operative histology and 5 cases (5.5%) by definitive fluid cytology. The remaining cysts were assessed using clinical and radiological evaluation, cytological characteristics, the “string sign” and clinical follow-up as standard reference. The diagnosis was made by the consensus-based decision of three experienced pancreatologists (IH, MH, and GG). We identified 52 mucinous cysts, including 33 intraductal papillary mucinous neoplasms (IPMNs), 12 mucinous cystadenomas and 7 adenocarcinomas. The remaining 39 cases were non-mucinous cysts, including 21 pseudocysts, 16 serous cystadenomas, 1 neuroendocrine tumor and 1 lymphoepithelial cyst (Table 2).

**TABLE 2:** Disease characteristics.

	Mucinous	Non-mucinous
Diagnosis based on	Number of cysts (%)	Number of cysts (%)
Follow up, Imaging	37 (71)	36 (92)
Postoperative histopathology	12 (23)	1 (3)
EUS guided FNA – definitive cytopathology	3 (6)	2 (5)

Diagnosis	Number of cysts (%)	Number of cysts (%)
IPMN	33 (63.5)	—
MCN	12 (23)	—
Adenocarcinoma	7 (13.5)	—
SCN	—	16 (41)
Pseudocyst	—	21 (54)
NET	—	1 (2.5)
Lymphoepithelial cyst	—	1 (2.5)

The median CEA level measured in the mucinous group was 449.5 ng/mL and 3 ng/mL (*p* < 0.0001, CI 95%) in the non-mucinous group. The median glucose level was shown to be 8.1 mg/dL in the mucinous group and 100.9 mg/dL in the non-mucinous group (*p* < 0.0001, CI 95%). CEA was above the cut-off level (192 ng/mL) in 36 of 52 mucinous cysts, resulting in a sensitivity of 69.2% for the differentiation of mucinous lesions. The intracystic glucose level was under the cut-off value (50 mg/dL) in 50 of 52 mucinous cysts, meaning a sensitivity of 96.2% in this regard. The specificity of CEA was shown to be 100%, while the specificity of glucose was 79.5%. The combination of the two methods (either CEA >192 ng/mL or glucose <50 mg/dL) improved the sensitivity to 100%, while the specificity was lower than that of CEA alone (79.5% vs. 100%). The results, including positive and negative predictive values, are presented in Table 3.

**TABLE 3:** Diagnostic performance of CEA, Glucose and CEA with Glucose combined.

​	Mucinous	Non-mucinous	*p-*value
CEA (Cutoff 192 ng/mL)			
CEA level, median (95% CI)	449.5 (1,587–58.23)	3 (16.3–3)	*<0.0001*
Elevated/non-elevated (n)	36/16	0/39	​
Glucose (Cutoff 50 mg/dL)			
Glucose level, median (95% CI)	8.1 (11.71–1.8)	100.9 (115.3–55.86)	*<0.0001*
Elevated/non-elevated (n)	2/50	31/8	​
CEA and glucose			
Either CEA elevated or glucose non-elevated	52/0	8/31	​
Amylase (Cutoff 250 IU/L)			
Amylase level, median (95%CI)	2,445 (1,011–8,825)	10,360 (173–52,800)	​
Elevated/non-elevated	40/12	23/16	​
​	CEA	Glucose	CEA and glucose
Sensitivity	69.2%	96.2%	100%
Specificity	100%	79.5%	79.5%
PPV	100%	86.2%	86.7%
NPV	70.9%	93.9%	100%

## Discussion

Differentiation between mucinous and non-mucinous pancreatic cysts remains challenging. Even if best practice according to the clinical guidelines is applied, only 72% of pancreatic cystic neoplasms are diagnosed correctly and adequate differentiation of mucinous cysts is made in 86% of cases [13]. Analysis of intracystic fluid obtained by EUS-FNA is an effective tool in improving the diagnostic accuracy for detecting mucinous cysts. Although current guidelines recommend the use of cytology and intracystic CEA for this purpose, CEA has a relatively low sensitivity (56%–67%) at a standardized cut-off value of 192 ng/mL, while the specificity has been shown to be 80%–95% in systematic reviews [8–10].

The optimal cut-off value of CEA is still unclear. A value of 20 ng/mL achieved the highest diagnostic accuracy with a sensitivity of 91% (95% Cl 88%–94%) and specificity of 85% (95% Cl 72%–93%) [14].

In 2013, a study of pancreatic cyst fluid analysis by Park et al. found glucose as a promising tool for identifying mucinous pancreatic cystic lesions [15]. In contrast to CEA, glucose level analysis has been well established in various biological fluids (serum, plasma, cerebrospinal fluid, urine) with good reproducibility, while the CEA assay is validated only in serum and its value in pancreatic cysts can vary across laboratories and assay kits [16]. Moreover, determining the level of glucose requires a significantly lower volume of intracystic fluid than the CEA assay (50 µL vs. at least 200 µL) [9].

The disadvantage of intracystic glucose measurement is that an infection of the cyst could cause, similar to mucinous cysts, a higher glucose consumption which could lead to a lower intracystic glucose level. Cysts with signs of infection on cytological or microbiological testing were therefore excluded from statistical analysis in our study.

Additionally, DNA markers, particularly mutation analysis of GNAS and KRAS, may be applied for identifying mucin-producing cysts with high sensitivity. According to the European Guideline on pancreatic cystic neoplasms, in cases in which the diagnosis is unclear and a change in diagnosis will alter management, analysis of these mutations may be considered. The minimal volume to perform molecular analysis is 0.2–0.5 mL, although in some samples the amount of DNA is insufficient to perform the analysis [17–19]. Furthermore, next-generation sequencing is costly and requires specialized experience, which limits the widespread use of this method.

Diagnostic performance of advanced EUS guided “through-the-needle” techniques, such as confocal laser endomicroscopy and Moray forceps biopsy is superior to intracystic biomarker measurements [20], however, the availability of these methods is limited to highly specialized centers.

A limitation of our study is that it is a single center series containing cysts with diagnosis mainly based on clinical follow-up, imaging and cytological features, with low proportion of cysts diagnosed by positive cytology (cystic adenocarcinoma or neuroendocrine tumor) or postoperative histology. Reliability of the final diagnosis in the remaining cases is equivocal. However, the assessed cysts in our study represent a wide spectrum of lesions with various sizes and morphology. Furthermore, a relatively small proportion of cysts require surgical resection in clinical practice and cytological testing rarely leads to a definitive diagnosis.

Another limitation is the mean follow up time of 921 days, which is a relatively short period in case of undefined pancreatic cystic lesions.

Our results are in accordance with the literature demonstrating an outstanding sensitivity of intracystic glucose in the differentiation of mucinous cysts. Our study shows that the highest sensitivity can be reached by using the combination of elevated intracystic CEA and lowered glucose levels. The specificity of the combined method proved to be higher than that of glucose, but lower than that of CEA alone.

## Conclusion

Current guidelines do not contain any recommendation regarding determination of intracystic glucose levels in the differentiation of mucinous versus non-mucinous pancreatic cysts. However, there have been several trials published in the last 10 years with conclusive results. In these, such as in our present study, glucose has been shown to be a cost effective, easily accessible, reliable cystic biomarker with a higher sensitivity in identifying mucinous pancreatic cysts compared to CEA. On the other hand, this study confirms the data in the literature regarding the low specificity of glucose, which diagnostic performance for pancreatic cystic lesions remains inferior to “through-the-needle” techniques, such as confocal laser endomicroscopy and Moray forceps biopsy [20]. However, glucose is more sensitive than CEA, and may be efficiently used as an additional biomarker in centers not equipped with “through-the-needle” techniques.

Nevertheless, further research and multicentric, randomized studies are needed to confirm the data regarding the usefulness of intracystic glucose measurement. If the results of these are congruent with the data from this and previously published studies, intracystic glucose assessment may be integrated into routine clinical practice and international guidelines.

## Data Availability

The raw data supporting the conclusions of this article will be made available by the authors, without undue reservation.
