# Correction: Diagnostic performance of intracystic carcinoembryonic antigen (CEA) versus glucose in differentiation of mucinous and non-mucinous pancreatic cysts

**DOI:** 10.3389/pore.2026.1612408

**Published:** 2026-03-27

**Authors:** György Gyimesi, Bánk Keczer, Péter Rein, Miklós Horváth, Ákos Szűcs, Tamás Marjai, Attila Szijártó, István Hritz

**Affiliations:** 1 School of Doctoral Studies, Semmelweis University, Budapest, Hungary; 2 Department of Gastroenterology, Spital Thurgau AG, Münsterlingen, Switzerland; 3 Department of Surgery, Transplantation and Gastroenterology, Semmelweis University, Budapest, Hungary; 4 Department of Surgery, Transplantation and Gastroenterology, Division of Interventional Gastroenterology, Semmelweis University, Budapest, Hungary

**Keywords:** pancreatic cyst, endosonography, FNA, intracystic CEA, intracystic glucose

There were mistakes in Table 3 as published. The values in the table are incorrect and do not correspond with the numbers in the abstract and in the text. The corrected Table 3 appears below.

**TABLE 3:** Diagnostic performance of CEA, Glucose and CEA with Glucose combined.

​	Mucinous	Non-mucinous	*p-*value
CEA (Cutoff 192 ng/mL)			
CEA level, median (95% CI)	449.5 (1,587–58.23)	3 (16.3–3)	*<0.0001*
Elevated/non-elevated (n)	36/16	0/39	​
Glucose (Cutoff 50 mg/dL)			
Glucose level, median (95% CI)	8.1 (11.71–1.8)	100.9 (115.3–55.86)	*<0.0001*
Elevated/non-elevated (n)	2/50	31/8	​
CEA and glucose			
Either CEA elevated or glucose non-elevated	52/0	8/31	​
Amylase (Cutoff 250 IU/L)			
Amylase level, median (95%CI)	2,445 (1,011–8,825)	10,360 (173–52,800)	​
Elevated/non-elevated	40/12	23/16	​
​	CEA	Glucose	CEA and glucose
Sensitivity	69.2%	96.2%	100%
Specificity	100%	79.5%	79.5%
PPV	100%	86.2%	86.7%
NPV	70.9%	93.9%	100%

The original article has been updated.

